# Diffusion Tensor Imaging Detects Acute and Subacute Changes in Corpus Callosum in Blast-Induced Traumatic Brain Injury

**DOI:** 10.1177/1759091420922929

**Published:** 2020-05-13

**Authors:** Palamadai N. Venkatasubramanian, Prachi Keni, Roland Gastfield, Limin Li, Daniil Aksenov, Sydney A. Sherman, Julian Bailes, Brian Sindelar, John D. Finan, John Lee, Julian E. Bailes, Alice M. Wyrwicz

**Affiliations:** 1 Center for Basic M.R. Research, Department of Radiology, NorthShore University HealthSystem, Evanston, Illinois, United States; 2 Department of Neurosurgery, NorthShore University HealthSystem, Evanston, Illinois, United States; 3 Department of Pathology, NorthShore University HealthSystem, Evanston, Illinois, United States

**Keywords:** blast injury, rat model, diffusion imaging, white matter, axonal injury, ultrastructure

## Abstract

There is a critical need for understanding the progression of neuropathology in blast-induced traumatic brain injury using valid animal models to develop diagnostic approaches. In the present study, we used diffusion imaging and magnetic resonance (MR) morphometry to characterize axonal injury in white matter structures of the rat brain following a blast applied via blast tube to one side of the brain. Diffusion tensor imaging was performed on acute and subacute phases of pathology from which fractional anisotropy, mean diffusivity, axial diffusivity, and radial diffusivity were calculated for corpus callosum (CC), cingulum bundle, and fimbria. Ventricular volume and CC thickness were measured. Blast-injured rats showed temporally varying bilateral changes in diffusion metrics indicating persistent axonal pathology. Diffusion changes in the CC suggested vasogenic edema secondary to axonal injury in the acute phase. Axonal pathology persisted in the subacute phase marked by cytotoxic edema and demyelination which was confirmed by ultrastructural analysis. The evolution of pathology followed a different pattern in the cingulum bundle: axonal injury and demyelination in the acute phase followed by cytotoxic edema in the subacute phase. Spatially, structures close to midline were most affected. Changes in the genu were greater than in the body and splenium; the caudal cingulum bundle was more affected than the rostral cingulum. Thinning of CC and ventriculomegaly were greater only in the acute phase. Our results reveal the persistent nature of blast-induced axonal pathology and suggest that diffusion imaging may have potential for detecting the temporal evolution of blast injury.

Blast-induced traumatic brain injury (bTBI) has been the signature injury in the conflicts in Iraq and Afghanistan because of the use of improvised explosive devices in terrorist attacks (Benzinger et al., 2009; Moore and Jaffee, 2010). It is estimated that approximately 10% to 20% of veterans returning from these conflicts have sustained bTBI in the theater, with the true prevalence of the long-term effects of bTBI suspected to be even higher (Ivanov et al. 2017). When an explosion occurs, blast injuries result from a pressure wave generated at a distance and transmitted through air. Damage to the nervous system is thought to occur through the traveling shock wave’s interaction with the brain (Nakagawa et al., 2011; Kucherov et al., 2012; Gupta and Przekwas, 2013; Elder et al., 2014). However, the injury directly related to the blast wave which is known as the primary blast injury is complicated by secondary, tertiary, and quaternary injury-causing mechanisms associated with blasts in the military setting. Secondary injury results from impact or penetration of blast-associated shrapnel, tertiary injury from falling debris or the soldier being thrown from vehicle resulting in shearing and impact injuries, and quaternary injury from thermal and toxic detonation products (Moore and Jaffee, 2010; Risling and Davidsson, 2012). As a result, neither the precise conditions that produce bTBI nor the development of pathology resulting from blast exposure are well understood. Also, clinical studies of bTBI in veterans have typically been conducted several months after blast exposure which represents the chronic stage of bTBI pathology (Mac Donald et al., 2011; Ivanov et al., 2017). Therefore, there is a critical need for understanding the progression of bTBI pathology in the acute and subacute stages in valid animal models of primary blast injury to develop diagnostic and therapeutic approaches.

Animals exposed to open-field explosive detonation have previously been used to study bTBI, but this model is not desirable because of the associated difficulty in dosimetry (Rubovitch et al., 2011; Risling and Davidsson, 2012). Alternatively, rat or mouse exposed to controlled blast waves produced by compressed gas in a shock tube has become an accepted animal model for studying neurotrauma resulting from primary blast injury (Elder et al., 2014). The range of neuronal and tissue damage caused in mice and rats by a single blast in a shock tube has been documented using a variety of histological techniques including immunohistochemistry and electron microscopy (EM; Ahlers et al., 2012; Goldstein et al., 2012; Sosa et al., 2013). Many of these studies implicate diffuse axonal injury in bTBI for which noninvasive neuroimaging approaches such as diffusion imaging are most suited.

Many nonblast TBI investigations, such as athletic injuries and automobile accidents, have focused on the corpus callosum (CC), a major white matter structure in the brain, because clinically axonal injury has been reported to be common in midline structures (Kraus et al., 2007; Wu et al., 2010a; Bazarian et al., 2012; Ling et al., 2012). While changes in anisotropy and diffusivity were detected in the CC when subjects were screened at different time points following the initial injury, these results were difficult to interpret as both increases and decreases in fractional anisotropy (FA) and mean diffusivity (MD) have been reported. Adult subjects with moderate to severe TBI had reduced FA in the CC compared with mild TBI (mTBI) subjects when screened 6 months post injury (Kraus et al., 2007). Consistent with this finding, children 7 to 17 years of age demonstrated lower FA and higher MD in the genu, body, and splenium of CC 3 months after injury (Wu et al., 2010a). On the other hand, neuroimaging within 21 days of injury showed elevated FA within the genu in mTBI patients along with significant decrease in radial diffusivity (RD; Ling et al., 2012). A study of high school athletes also reported increases in FA and decreases in MD in the body of CC and other white matter structures post athletic season when compared with preseason measures (Bazarian et al., 2012). Animal studies of TBI employing cortical controlled impact injury (CCI) or fluid percussion injury models have reported reduced FA along with altered diffusivities in the injured CC (Mac Donald et al., 2007a, 2007b; Laitinen et al., 2015; Harris et al., 2016; Wright et al., 2016). Previous studies of blast injury in animal models, however, have looked predominantly at pathology in the cortex and the hippocampus, and therefore, no details were provided about changes in the CC (Rubovitch et al., 2011; Goldstein et al., 2012; Budde et al., 2013; Sosa et al., 2013; Kamnaksh et al., 2014). Thus, there is a need to characterize blast-induced axonal injury in white matter structures using neuroimaging to understand how the diffusion metrics in major white matter structures change with the development and progression of injury over a given time frame.

In the current study, we used *in vivo* diffusion MR imaging and MR morphometry to characterize alterations to major white matter tracts in the rat brain at acute and subacute time points following injury induced by a single blast applied via a blast tube. Diffusion tensor imaging (DTI) yielded measures of anisotropy (FA) and diffusivity (MD, axial diffusivity [AD], and RD) in the CC, cingulum bundle, and fimbria, and thickness of the CC was measured from FA images. These two measures are considered to yield different neuropathological end points: DTI reveals axonal microstructure and pathology, whereas gross morphometry reflects axonal loss. In addition, ventricular area was obtained from T_2_-weighted images. Ventricular volumetry examines a critical brain homeostasis involving the ventricular system and cerebrospinal fluid (CSF) which has important functions such as providing intracranial buoyancy, absorbing shocks in the event of an impact, removal of metabolic waste, and transporting critical nutrients and hormones (Xiong et al., 2014). Further, to understand the association between diffusion changes and underlying pathology, we have compared the CC ultrastructure of blast injured and control rats at the subacute time point. EM of the injured brains provides the structural basis for explaining the observed diffusion changes.

## Materials and Methods

### Animal Specifications

This study used 3- to 6-month-old male Sprague Dawley rats (Charles River Laboratories, Wilmington, MA) weighing between 200 and 250 g. All animals were housed at the NorthShore University HealthSystem animal facility with appropriate feed and water and maintained on normal night/day cycles. The experimental protocol was approved by the NorthShore Institutional Animal Care and Use Committee.

### Blast Methods

Rats were anesthetized with isoflurane (4% for induction and 1% to 3% for maintenance) during the blast and received 0.05 mg/kg buprenorphine for post exposure analgesia. Each animal was fitted with ear plugs to protect the ears, placed inside an aluminum tube lined with foam to protect the torso and positioned in ventral recumbency, perpendicular to the axis of the blast tube opening to receive a right lateralized blast. The shock tube apparatus used to generate the bTBI has been validated in previous studies (Effgen et al., 2012; Panzer et al., 2012; Gullotti et al., 2014).

All animals’ heads were positioned about 2 cm from the exit of the driven section. The driver section was separated from the driven section by a stack of mylar membranes (McMaster Carr, Elmhurst, IL) and pressurized with helium. At a pressure determined by the thickness of the mylar membranes, the membranes ruptured, and a shock wave traveled down the driven section. Three piezoresistive pressure transducers (8530B-200, Endevco Meggitt, Irvine, CA) are mounted in the wall of the blast tube at the exit to record blast overpressure and duration.

Rats in the bTBI group received a blast characterized by mean peak pressure 417.79 kPa, mean duration 1.10 ms, and mean impulse 182.34 kPa ms. Control animals (*n* = 4) were prepared identical to the experimental group (*n* = 5) but did not receive the blast injury. Rats were imaged 1 day and 14 days after the blast.

### Diffusion Tensor Imaging

DTI was performed on a Bruker Biospec 9.4T MR imager (Billerica, MA) using a volume coil for excitation and a rat brain surface coil for signal detection. Rats were anesthetized with isoflurane (3% for induction and 1.5% to 2.0% for maintenance) during imaging. Respiration was monitored, and image acquisition was gated to the respiratory signal. Following the acquisition of localizer images, magnetic field homogeneity was optimized on the brain using the field map method (Miyasaka et al., 2006). Multislice DTI was acquired using a segmented echo planar imaging-DTI pulse sequence with field of view 1.89 cm × 1.30 cm, matrix size of 140 × 96 for spatial resolution of 135 μm × 135 μm in plane, 1.0 mm slice thickness, and 6 axial slices consisting of the genu, body, and splenium of the CC (2 slices per each CC region). The following imaging parameters were used: recycle time/echo delay time: 1,500 ms/19.1 ms, 16 segments, duration of diffusion gradients: 3 ms, delay between diffusion gradients: 7 ms, 30 gradient directions, one *b* value = 852 s/mm^2^. Maps of FA, Trace, and diffusivity along the *x*, *y*, and *z* axes were generated by processing the diffusion-weighted images using ParaVision 5.1 software (Bruker Biospin, Billerica, MA).

### Image Analysis

**Figure 1.**
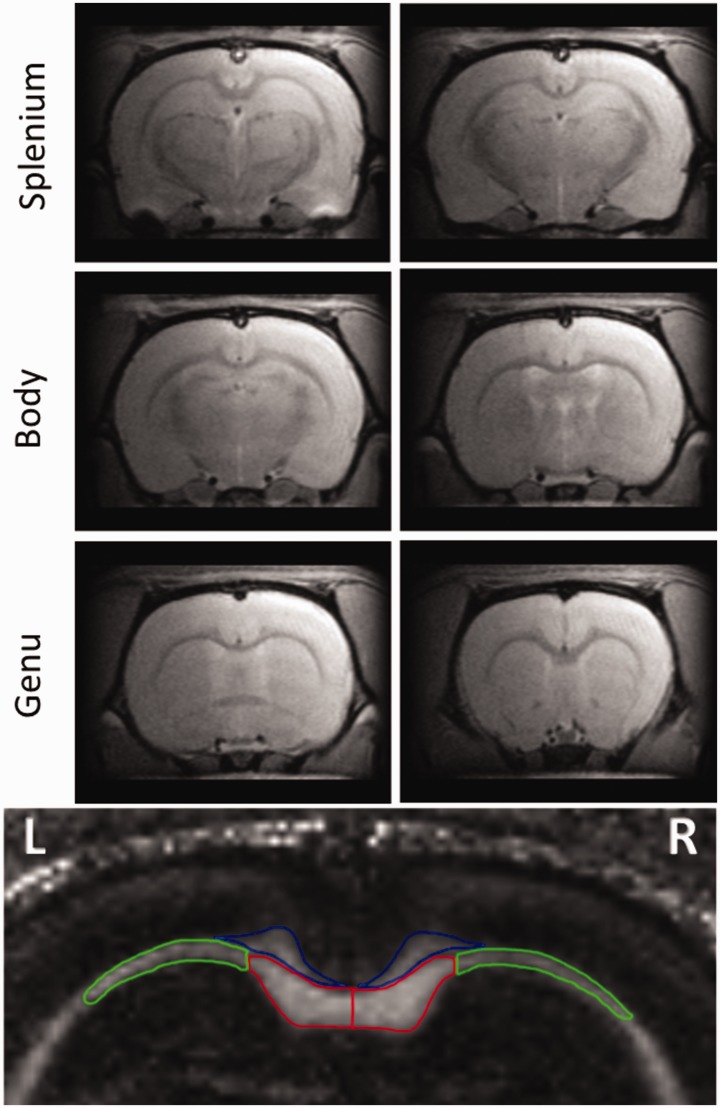
T_2_-Weighted MR Images of the Axial Slices That Were Used to Measure Fractional Anisotropy (FA), Mean Diffusivity (MD), Axial Diffusivity (AD), and Radial Diffusivity (RD) in White Matter Structures. Each of the three CC regions, namely, genu, body, and splenium, was present in two slices as shown. The following regions of interest used for diffusion measurements are indicated in color on an FA map at the bottom: right and left medial (red) and lateral (green) CC, cingulum bundle (blue). Diffusion metrics of the fimbria was measured from slices containing CC body.

DTI data from the six axial brain slices were analyzed separately and then averaged into three CC regions: genu, body, and splenium (Figure 1). The following regions of interest were manually delineated on both left and right hemispheres of the brain on the parameter images for each slice: medial CC, lateral CC, cingulum, and fimbria. The investigators performing the delineation were blinded to the groups. Reproducibility of delineation was verified between two independent operators, and the difference was found to be negligible. FA and MD were measured from the respective maps. AD and RD were calculated from the measured values of diffusivities in *x*, *y*, and *z* directions. Based on the directionality of white matter tracts in the CC and cingulum, axial and radial directions were defined for each region.

CC width was measured in FA maps of slices containing the genu. FA maps provided better contrast between CC and surrounding regions than T_2_-weighted images and therefore resulted in more accurate morphological measurements. Measurements were taken at three different locations in the medial part and averaged. CC widths are expressed in pixel units.

### Transmission Electron Microscopy

A separate group of rats exposed to the blast (*n* = 3) and sham controls (*n* = 3) were used for ultrastructural analysis of the genu. Rats were euthanized 17 days post blast via cardiac perfusion. The animals were perfused transcardially with 0.9% saline followed by 2% paraformaldehyde and 3% glutaraldehyde. Dissected brains were postfixed in fixative overnight. Sections of the CC genu were prepared using the protocol described previously (Mierzwa et al., 2015). EM was performed on an FEI Tecnai Spirit G2 Transmission Electron Microscopy scanner at the Center for Advanced Microscopy at Northwestern University.Inner axon radius and outer fiber radius were measured from EM images of myelinated axons from which *g*-ratio (ratio between inner and outer radii) as well as myelin thickness were calculated. Measurements from 20 separate myelinated axons from each animal were averaged for comparison of *g*-ratio and myelin thickness between bTBI and sham groups.

### Statistical Analysis

Means of measured values ± standard deviation were used for all statistical analysis. Statistical analysis of diffusion data was performed using STATISTICA 7 (StatSoft, Tulsa, OK). Factorial analysis of variance (ANOVA) was used to evaluate diffusion changes induced by blast using the following four factors: group (sham, blast); side (left, right); time point (Day 1 [D1], Day 14 [D14]); and location (genu, body, and splenium for medial and lateral CC; rostral, mid, and caudal for cingulum bundle). Post hoc Fisher least significant difference was used to compare diffusion changes that were dependent on any of the factors. Changes in ventricular area were evaluated using factorial ANOVA with three factors: group, side, and time point, followed by post hoc comparison. Standard two-tailed *t* tests with unequal variances were performed to compare the CC thickness of blast-injured rats to sham-treated animals at each time point and for EM data analysis. Statistical significance is defined as *p* < .05 for all data.

## Results

### Gross Anatomical Changes

All animals recovered normally from anesthesia administered during blast and subsequent imaging sessions. MR images showed no gross tissue alterations, intraventricular hemorrhages, or hemorrhages in the brains of rats exposed to the blast.

### Diffusion Imaging

Rats exposed to sham injury did not show any difference between D1 and D14 in the diffusion metrics of the structures examined. A complex pattern of diffusion changes was observed in the major axonal structures of bTBI animals which are discussed in detail later. To simplify the complex spatial and temporal evolution of neuropathology, only the following comparisons were made: (a) in the acute phase (D1), diffusion metrics in the bTBI group are compared with the sham treatment group, and (b) in the subacute phase (D14), comparisons are made between D14 and D1 values within the bTBI group.

### Fractional Anisotropy

**Figure 2.**
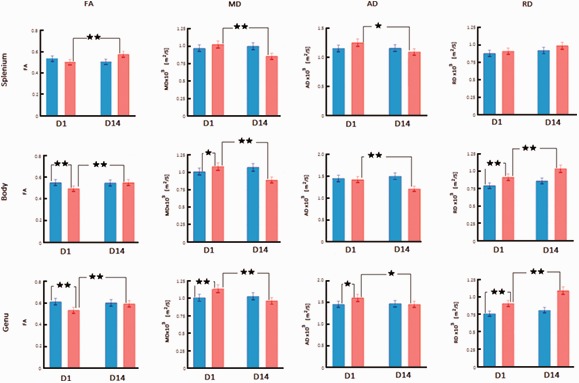
Diffusion Imaging Changes in Medial Right Hemisphere CC Regions After bTBI. FA, MD, AD, and RD are compared between sham-treated rats (blue bars) and blast-injured rats (red bars) at 1 day (D1) and 14 days (D14) after exposure to blast. Mean ± standard deviation of diffusion metrics was measured from regions representing medial CC in the splenium, body, and genu as shown in Figure 1. Significant changes obtained from Fisher post hoc analysis are marked at the level of *p* < .05 (*) and *p* < .01 (**). Only changes for sham versus bTBI at D1 and D1 versus D14 within the bTBI group are indicated. FA = fractional anisotropy; MD = mean diffusivity; AD = axial diffusivity; RD = radial diffusivity; D1 = Day 1; D14 = Day 14.

In the medial CC, there were significant main effects of only the factors of time point, *F*(1, 96)  = 20.39, *p* < .0001, and location, *F*(2, 96)  = 34.20, *p* < .0001. Interactions of group and time point, *F*(1, 96) = 31.19, *p* < .0001, as well as group and location, *F*(2, 96) = 9.18, *p* = .0002, were significant. Post hoc analysis showed that at D1, FA decreased significantly in the medial genu and body of bTBI rats relative to sham-treated animals on the right hemisphere (*p* < .01; Figure 2) and in the medial genu on the left hemisphere (*p* < .01; Supplementary Table 1). At D14, FA increased significantly in all three CC locations on the right (*p* < .01) and left hemispheres (*p* < .05).

**Figure 3.**
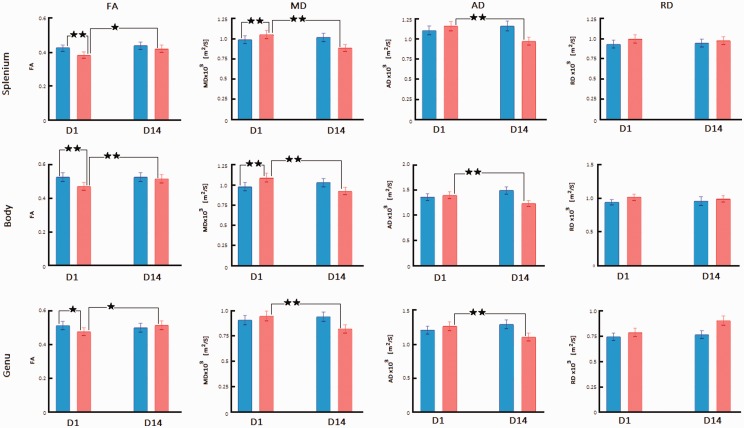
Diffusion Imaging Changes in the Lateral Right Hemisphere CC Regions After bTBI. FA, MD, AD, and RD are compared between sham-treated rats (blue bars) and blast-injured rats (red bars) at 1 day (D1) and 14 days (D14) after exposure to blast. Mean ± standard deviation of diffusion metrics was measured from regions representing the lateral CC in the splenium, body, and genu as shown in Figure 1. Significant changes obtained from Fisher post hoc analysis are marked at the level of *p* < .05 (*) and *p* < .01 (**). Only changes for sham versus bTBI at D1 and D1 versus D14 within the bTBI group are indicated. FA = fractional anisotropy; MD = mean diffusivity; AD = axial diffusivity; RD = radial diffusivity; D1 = Day 1; D14 = Day 14.

In the lateral CC, there were significant main effects of side, *F*(1, 96) = 16.78, *p* < .0001, group, *F*(1, 96) = 23.13, *p* < .0001, time point, *F*(1, 96) = 21.63, *p* < .0001, and location, *F*(2, 96) = 129.94, *p* < .0001. Interactions of group and time point, *F*(1, 96) = 20.55, *p* < .0001, as well as group and location, *F*(2, 96) = 3.68, *p* = .0289, were significant. Post hoc analysis showed that at D1, FA decreased significantly in bTBI rats relative to sham-treated animals in all three CC locations on the right (*p* < .05 in the genu and < .01 in the body and splenium) and in the body (*p* < .01) and splenium (*p* < .01) on the left hemisphere (Figure 3 and Supplementary Table 1). At D14, FA increased significantly in all three CC regions on the right and left hemispheres (*p* < .05).

**Figure 4.**
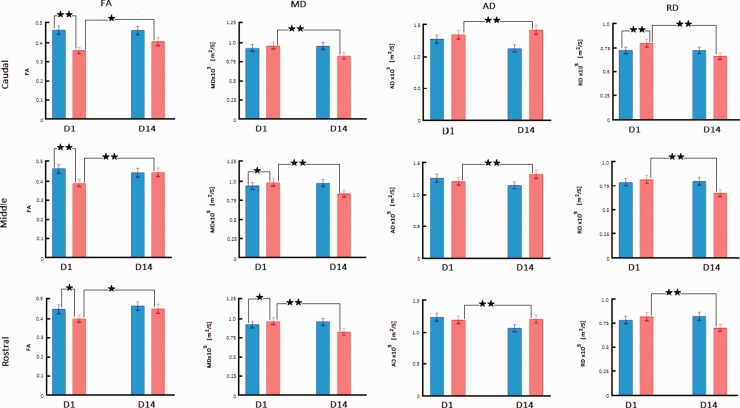
Diffusion Imaging Changes in the Cingulum Bundle Regions After bTBI. FA, MD, AD, and RD are compared between sham-treated rats (blue bars) and blast-injured rats (red bars) at 1 day (D1) and 14 days (D14) after exposure to blast. Mean ± standard deviation of diffusion metrics was measured from regions representing the cingulum bundle in slices containing splenium (caudal cingulum), body (middle cingulum), and genu (rostral cingulum). Each cingulum region is represented by two axial slices shown in Figure 1. Significant changes obtained from Fisher post hoc analysis are marked at the level of *p* < .05 (*) and *p* < .01 (**). Only changes for sham versus bTBI at D1 and D1 versus D14 within the bTBI group are indicated. FA = fractional anisotropy; MD = mean diffusivity; AD = axial diffusivity; RD = radial diffusivity; D1 = Day 1; D14 = Day 14.

In the cingulum bundle, there were significant main effects of only group, *F*(1, 96) = 65.30, *p* < .0001, and time point, *F*(1, 96) = 10.84, *p* = .0014. Interactions of group and time point, *F*(1, 96) = 13.60, *p* = .0004, as well as group and location, *F*(2, 96) = 4.46, *p* = .0141, were significant. Post hoc analysis showed that at D1, FA decreased in all three cingulum locations on the right (*p* < .05 at rostral, and < .01 at middle and caudal cingulum) and left (*p* < .05 at rostral, and < .01 at middle and caudal cingulum) hemispheres of bTBI rats relative to sham-treated animals (Figure 4 and Supplementary Table 1). At D14, FA increased significantly in all cingulum locations (*p* < .05).

### Mean Diffusivity

In the medial CC, there were significant main effects of group, *F*(1, 96) = 6.23, *p* = .0142, time point, *F*(1, 96) = 50.08, *p* < .0001, and location, *F*(2, 96) = 17.26, *p* < .0001. Interactions of group and time point, *F*(1, 96) = 123.60, *p* < .0001, as well as group and location, *F*(2, 96) = 8.02, *p* = .0006, were significant. Post hoc analysis showed that at D1, MD increased significantly in the right medial genu (*p* < .01) and body (*p* < .05), and left genu (*p* < .05) of bTBI rats relative to sham-treated animals (Figure 2 and Supplementary Table 1). At D14, MD decreased significantly in all three regions of the medial CC on the right and left hemispheres (*p* < .01).

In the lateral CC, there were significant main effects of group, *F*(1, 96) = 14.73, *p* = .0002, time point, *F*(1, 96) = 79.76, *p* < .0001, and location, *F*(2, 96) = 79.14, *p* < .0001. Interactions of group and time point, *F*(1, 96) = 211.99, *p* < .0001, as well as group and location, *F*(2, 96) = 4.09, *p* = .0197, were significant. Post hoc analysis showed that at D1, MD increased significantly in bTBI rats relative to sham-treated animals at the following lateral CC locations: right body and splenium (*p* < .01), and all regions of left lateral CC (*p* < .05; Figure 3 and Supplementary Table 1). At D14, MD decreased significantly in all CC regions on the right and left hemispheres (*p* < .01).

In the cingulum bundle, there were significant main effects of group, *F*(1, 96) = 41.12, *p* < .0001, and time point, *F*(1, 96) = 74.28, *p* < .0001. Interaction of group and time point, *F*(1, 96) = 185.91, *p* < .0001, only was significant. Post hoc analysis showed that at D1, MD increased significantly in bTBI rats relative to sham-treated animals at the following cingulum locations: rostral and middle cingulum on the right (*p* < .05), and all regions on the left (*p* < .05; Figure 4 and Supplementary Table 1). At D14, MD decreased significantly in all CC regions on the right and left hemispheres (*p* < .01).

### Axial Diffusivity

In the medial CC, there were significant main effects of time point, *F*(1, 96) = 13.52, *p* = .0004, and location, *F*(2, 96) = 95.53, *p* < .0001. Interactions of group and time point, *F*(1, 96) = 28.65, *p* < .0001, as well as group and location, *F*(2, 96) = 10.97, *p* < .0001, were significant. Post hoc analysis showed that at D1, AD increased significantly in the right and left medial genu of bTBI rats relative to sham-treated animals (*p* < .05; Figure 2 and Supplementary Table 1). At D14, AD decreased significantly in all three regions of the medial CC on right and left hemispheres in the bTBI group (*p* < .05).

In the lateral CC, there were significant main effects of group, *F*(1, 96) = 13.01, *p* = .0005, time point, *F*(1, 96) = 8.78, *p* = .0038, and location, *F*(2, 96) = 111.85, *p* < .0001. Interactions of side and group, *F*(1, 96) = 5.60, *p* = .01996, as well as group and time point, *F*(1, 96) = 102.53, *p* < .0001, were significant. Post hoc analysis showed that at D1, AD decreased significantly in all three CC locations on the left hemisphere of bTBI rats relative to sham-treated animals (*p* < .05), whereas there were no changes in the right hemisphere (Figure 3 and Supplementary Table 1). At D14, AD decreased significantly in all lateral CC regions compared with D1 values of the bTBI animals (*p* < .01).

In the cingulum bundle, there were significant main effects of group, *F*(1, 96) = 53.11, *p* < .0001, time point, *F*(1, 96) = 6.32, *p* = .0136, and location, *F*(2, 96) = 29.25, *p* < .0001. Interactions of group and time point, *F*(1, 96) = 81.73, *p* < .0001, as well as group and location, *F*(2, 96) = 8.13, *p* = .0005, were significant. Post hoc analysis showed that AD was unchanged in all regions at D1 but increased significantly in all locations at D14 (*p* < .01; Figure 4 and Supplementary Table 1).

### Radial Diffusivity

In the medial CC, there were significant main effects of group, *F*(1, 96) = 128.64, *p* < .0001, and time point, *F*(1, 96) = 56.43, *p* < .0001. Interactions of group and time point, *F*(1, 96) = 12.10, *p* = .0008, as well as group and location, *F*(2, 96) = 13.38, *p* < .0001, were significant. Post hoc analysis showed that at D1, RD increased significantly in the genu and body on right and left medial CC of bTBI rats relative to sham-treated animals (Figure 2 and Supplementary Table 1; *p* < .01). At D14, RD in the bTBI group increased significantly in the right genu and body and all three regions of the left medial CC relative to their D1 values (*p* < .01).

In the lateral CC, there were significant main effects of the factors time point, *F*(1, 96) = 5.80, *p* = .0179, and location, *F*(2, 96) = 9.86, *p* = .0001. There were no significant interactions of factors. Post hoc analysis showed no significant changes in RD in the lateral CC at any time point (Figure 3 and Supplementary Table 1).

In the cingulum bundle, there were significant main effects of group, *F*(1, 96) = 9.53, *p* < .0026, time point, *F*(1, 96) = 80.73, *p* < .0001, and location, *F*(2, 96) = 15.25, *p* < .0001. Interactions of group and time point, *F*(1, 96) = 74.09, *p* < .0001, as well as group and location, *F*(2, 96) = 5.92, *p* = .0038, were significant. Post hoc analysis showed that at D1, RD significantly increased in the bTBI rats compared with control animals on both right and left caudal cingulum (*p* < .01; Figure 4 and Supplementary Table 1). At D14, RD in the bTBI group decreased from D1 values at all cingulum locations in both hemispheres (*p* < .01).

For an easier comparison of the different patterns of changes, Supplementary Table 2 summarizes the increases and decreases of diffusion metrics in CC and cingulum fibers of the right hemisphere of bTBI rats at different time points.

Diffusion metrics were measured in the fimbria from the slice locations corresponding to the body of CC. FA showed main effect of group, *F*(1, 32) = 31.315, *p* < .0001, and interaction of group and time point, *F*(1, 32) = 25.989, *p* < .0001; MD showed main effect of time point, *F*(1, 32) = 20.15, *p* < .0001, and interaction of group and time point, *F*(1, 32) = 61.79, *p* < .0001. At D1, FA and MD increased significantly in bTBI rats (*p* < .01), and at D14, FA increased (*p* < .05) while MD decreased (*p* < .01) from D1 values. AD and RD components were not separated in the fimbria as the directionality components of the individual diffusivities were not along one of the three major axes.

### MR Morphometry

**Figure 5.**
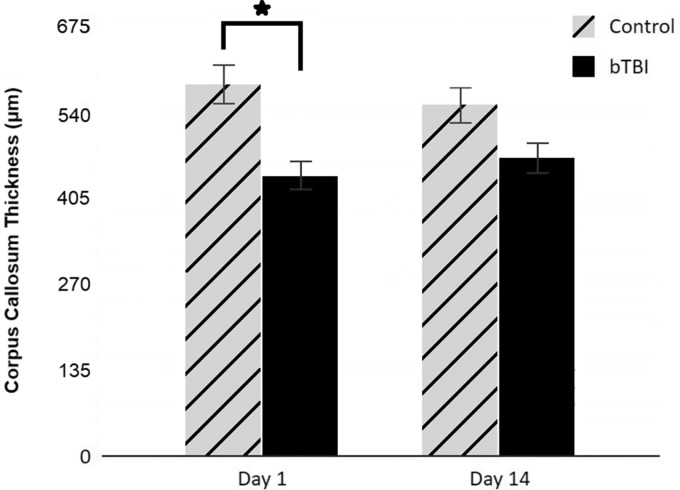
Thinning of Corpus Callosum Was Detected by MR Imaging Following Blast Injury. Thickness of CC at the level of the genu was measured in axial FA images from DTI. Measurements taken from three locations (at midline and at 1.2 mm lateral on either side) were averaged and expressed as mean ± standard deviation. Bar graph compares CC thickness in blast-injured animals to the control values at D1 and D14 (*p* < .05). bTBI = blast-induced traumatic brain injury; D1 = Day 1; D14 = Day 14.

Thickness of CC was compared between control and blast-injured rats (Figure 5). The results indicate a significantly reduced CC thickness in blast-injured rats on D1 (*p* < .05) and recovery to near control thickness on D14. Ventricular changes in rats exposed to blast injury were examined by measuring the area of lateral and third ventricles from T_2_-weighted A0 images that were part of the diffusion data set. ANOVA showed main effects of group, *F*(1, 32) = 114.12, *p* < .0001, and time point, *F*(1, 32) = 6.65, *p* = .0147. Post hoc analysis revealed that at D1, ventricular area increased in both left and right hemispheres of bTBI rats (*p* < .01) relative to sham-treated rats and remained elevated at D14 (*p* < .05). Average ventricular enlargements were 151.9% at D1 and 109.9% at D14.

### EM of the CC

**Figure 6.**
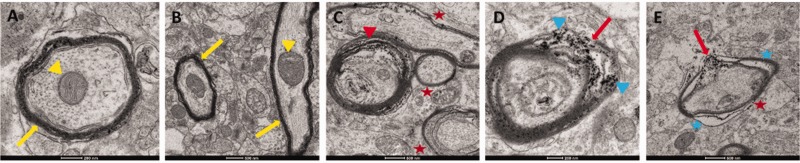
Electron Microscopy Images Reveal Alterations in CC Fibers 17 Days After bTBI. A: Transverse section of a myelinated axon in the genu from a sham-treated rat. Tightly organized myelin sheath (yellow arrow) around the fiber and loosely packed neurotubules within the fiber are characteristic of a normal myelinated axon in the CC. B: Longitudinal section of an axon in the genu from a sham-treated rat shows uniform, intact myelination along the length of the fiber (yellow arrow) and well-organized neurofilaments within it. Transverse section of a nearby axon shows the characteristic features of a normal myelinated axon seen in A. Mitochondria within these axons appear normal (yellow arrowheads). C to E: Axons in the genu from blast-injured rats show extensive degeneration. Separation of the myelin sheath from the fiber (C, red arrowhead), damage to myelin sheath (D, E, red arrows), accumulation of debris (D, blue arrowhead) most likely from degenerating myelin sheath, swollen axons, breakdown of cytoskeletal filaments, severe deformity with regions of edema (E, blue asterisk), and myelin thinning (C, E, red asterisks) are widespread in the injured rat CC. Similar ultrastructural changes were seen in all blast-injured rats. All control rat EM resembled the sham-treated rat images presented here.

To decipher the cytological basis for diffusion metric changes in blast-injured CC, we compared the ultrastructure of axons in the genu of blast-injured and sham rats. Analysis of ultrastructure using EM revealed alterations in the axons of the genu 17 days after exposure to a single blast indicating compromised white matter integrity. Normal myelinated axons with a compact myelin sheath and well-organized microtubules and neurofilaments were seen in the images of control brains (Figure 6A, B). In contrast, demyelination is clearly evident in bTBI CC (Figure 6C to E) with microstructural abnormalities in the bTBI brains including thinning of myelin sheath, separation of myelin layers from the fiber, fragmentation of the myelin sheath, edema, and accumulation of debris from degenerating myelin sheath. To further quantify changes in myelination, *g*-ratios were calculated from EM images. A significant increase in *g*-ratio was observed in bTBI brains (0.69 ± 0.02 in sham injury vs. 0.83 ± 0.06 in bTBI; *p* = .0037) as a result of 25% increase in axon caliber (2.0 µm vs. 2.5 µm) and 46.7% decrease in myelin sheath thickness (0.9 µm vs. 0.48 µm).

## Discussion

### Temporal Evolution of bTBI

Temporal evolution of diffusion metrics was examined in three major white matter structures in the brain, the CC, cingulum bundle, and the fimbria, to understand the evolving pathology following exposure to a single blast. The CC forms the largest commissural white matter bundle in the brain, and its role is essential to the integration of the information between left and right cerebral hemispheres. The cingulum bundle is a prominent white matter tract that extends longitudinally above the CC and is an important connection within the limbic system. Also part of the limbic system and housed within the temporal horn of the lateral ventricle, the fimbria covers the rostral portion of the hippocampus as its afferent and efferent fibers. In the acute phase following bTBI (D1), all three white matter bundles of the brain display a complex pattern of region-dependent pathologies that include axonal injury, vasogenic edema, and demyelination. Two weeks after the initial blast, we detected persistent, spatially widespread axonal injury in these regions.

Acute phase changes in the medial genu include lower FA, higher MD, and higher AD and RD. Lower FA is considered to indicate axonal injury and is typically accompanied by elevated MD (Basser and Jones, 2002). The combination of lower FA, higher MD, and increases in both RD and AD in acute bTBI suggests the presence of vasogenic edema secondary to axonal injury that surrounds the CC fibers (Lin et al., 2016).

In the subacute phase, the most prominent changes in the CC are increase in FA accompanied by decrease in MD. If considered alone, increase in FA that follows an earlier decrease would be interpreted as recovery from axonal injury. However, changes in the diffusivity components indicate otherwise: lower AD and elevated RD reveal that axonal pathology persists 2 weeks after exposure to blast (Winklewski et al., 2018). Lower AD in combination with elevated FA and lower MD also represents cytotoxic edema (Lin et al., 2016). Elevated RD represents demyelination that was confirmed by ultrastructural imaging of the genu.

Axons at various stages of demyelination and debris from degenerating myelin sheath are clearly evident in EM images of the genu from bTBI rats, revealing the cellular basis for the increase in RD in the CC. Increases in *g*-ratio and decreases in myelin thickness provide a quantitative measure of demyelination. The *g*-ratio is important for white matter efficiency and conduction fidelity, and maintaining an optimal *g*-ratio is critical for reliable signal transduction in white matter (Rushton, 1951; Chomiak and Hu, 2009). In healthy rodents, the *g*-ratio ranges from 0.6 to 0.8 (Guy et al., 1989; Arnett et al., 2001), and in the human CC, it is 0.69 (Berman et al., 2018), which matches the *g*-ratio measured in sham-injured rats in our study. In contrast, *g*-ratio is 0.83 in blast-injured rats more than 2 weeks after the initial injury which indicates the persistent nature of bTBI. Swollen axons, deformed axons, and edema within the damaged axon seen in our EM images lend support to the conclusion based on diffusion metrics that cytotoxic edema and axonal pathology persist more than 2 weeks after blast injury. Demyelination of the genu in bTBI may have mental health implications. The CC is a key area for studying severe mental illness. Cases of major depressive disorder had significantly decreased mean myelin cross-sectional area in the splenium than in controls (Williams et al., 2019). Injury to the CC may have other behavioral implications also. Because the fibers of CC overlying the ventricles are associated with motor skill learning (Yu et al. 2017), axonal injury in our bTBI rat model may affect motor coordination. Prolonged motor abnormalities have been reported in a mouse model of blast injury with multifocal axonal injury (Koliatsos et al. 2011). Persistent memory deficits and changes in hippocampal FA have been reported in primary blast injury in the rat (Kovesdi et al. 2012; Budde et al., 2013). However, there were no detectable changes in the hippocampus in our diffusion measurements.

Prominent acute phase diffusion changes in the cingulum bundle consist of lower FA and elevated MD which suggests axonal injury. Increase in FA and decrease in MD in the subacute phase might suggest cytotoxic edema. Alternatively, increase in AD seen in the cingulum in the subacute phase could also indicate recovery from axonal injury. This is supported by the observed decrease in RD which most likely indicates remyelination of the cingulum axons in contrast to the demyelination of CC fibers. This difference in axonal pathology between CC and cingulum fibers strongly suggests that blast forces act differently on fibers that are parallel to the direction of the force than on fibers that are perpendicular to its direction. DTI investigations of automobile accident-related TBI in children and adolescents have reported increased FA and reduced apparent diffusion coefficient (ADC) in the cingulum during the acute stage reflecting cytotoxic edema and lower FA and elevated ADC in the chronic stage indicating axonal injury (Wilde et al., 2010; Wu et al., 2010b). Pathology seen in the cingulum bundle may have implications in the emergence of neuropsychiatric consequences following bTBI (Lepage et al., 2018). Mice exposed to repetitive blast injury displayed damage to cingulate cortex and impaired social behavior (Yu et al., 2017). Diffusion changes in the fimbria, namely increases in both FA and MD in the acute phase followed by further increase in FA but decrease in MD, suggest that injury is persistent in this white matter structure also. The pattern of diffusion changes seen in the fimbria, however, is unusual and difficult to explain by known neuropathological patterns in the absence of AD and RD measurements. There has been no previous report on the effects of bTBI on the fimbria.

Although the blast wave was applied from the right side to the rat head, there was absence of laterality in the resulting pathology. The magnitude of changes in all the diffusion metrics was nearly identical in corresponding white matter structures on both ipsilateral and contralateral sides. In this regard, bTBI does not cause focal injury such as fluid percussion or CCI models of TBI in which unilateral vulnerability of the brain occurs. Following fluid percussion injury, rats showed changes in FA and MD only in the ipsilateral CC at acute and chronic time points (Laitinen et al., 2015; Harris et al., 2016; Wright et al., 2016). Unilateral reduction in anisotropy revealing injury only on the ipsilateral side has been reported in both rat and mouse models of CCI (Mac Donald et al., 2007b; Harris et al., 2016). Consistent with our results, Goldstein et al. (2012) have reported extensive bilateral neuropathology in cortical and hippocampal neurons in mice exposed to a single blast impacting the left side. In contrast to this, a previous DTI study of primary blast injury in rats in which animals were positioned off-axis and about 6 inches from the shock tube opening found greater decreases in FA on the ipsilateral side (Budde et al., 2013), which emphasizes the need for uniformity among models when comparing results of bTBI studies.

We found that the severity of injury was spatially dependent along the medial–lateral and rostrocaudal directions. Medial CC fibers show greater change in diffusion metrics compared with lateral CC at D1 indicating that injury was more severe closer to the midline. Further, changes in the medial genu were of a higher magnitude than those in the medial body with the splenium least affected, strongly suggesting that the genu is most vulnerable to blast and injury severity decreases in a rostrocaudal direction. This is in contrast to the reported selective vulnerability of the splenium in a rat model of fluid percussion injury (Reeves et al., 2012). The rostrocaudal differences in the CC fibers that we find are similar to what has been reported in patients with differing severities of brain injury (Rutgers et al., 2008). Compared with controls, patients with mTBI had reduced FA and increased ADC only in the genu, while patients with moderate and severe TBI also had reduced FA in the splenium. Spatial differences in response to bTBI may have their origin in the axon phenotype in each CC region in the rat brain. Light and electron microscopic analyses have shown differences in the diameter, packing, and myelination of CC fibers in the genu, body, and splenium, which are also dependent on gender and species (Aboitiz et al., 1992; Reeves et al., 2005; Wahl et al., 2007; Al Qattan et al., 2019). Differences in fiber direction between medial and lateral CC are likely to influence the relative changes in diffusion metrics between those CC regions. Diffusion changes in the cingulum bundle do not appear to be spatially dependent in the rostrocaudal or left–right directions.

### MR Morphometry

Occurrence of CC thinning appears to vary among TBI models and human TBI. In our case, thinning of the CC occurred only in the acute phase of blast injury in rats, unlike in children who showed persistent volumetric decreases in CC subregions between 3 and 18 months after moderate to severe injury which can be considered as the chronic phase of TBI (Wu et al., 2010a). Recovery of CC thickness in the subacute phase most likely suggests that there is no axonal loss in our bTBI model. In contrast, impact injury in the mouse showed CC thinning only in some mice (Mierzwa et al., 2015), suggesting that acute-stage pathology in impact injury is different from that of bTBI. The pathology associated with CC thinning and its relationship to changes in diffusion metrics are at present unclear. A limitation to our MR image-based CC morphometry is that the differences in thickness measured may lack precision because of the large pixel size and the small number of pixels that comprise the CC.

Enlargement of ventricles following bTBI appears to be sensitive to the number of days post injury, suggesting that this measurement may have utility as a biomarker to assess the stage of bTBI. The temporal pattern of increased ventricular volume and thinning of the CC appear to be parallel; however, additional investigations are needed to establish a causal relationship between the two. Ventricular enlargement following head injury has been reported in patients (Kim et al., 2015) and in animal models of TBI (Xiong et al., 2014). However, detailed studies of the ventricular system including measurements of CSF volume, pressure, and flow in TBI patients are lacking. In animal models, injury-induced ventricular system pathology resolves completely by 30 days post injury. Using a particle tracking technique, it was shown that diminished CSF flow was responsible for ventricular enlargement in a mouse model of fluid percussion injury (Xiong et al., 2014). Alternatively, it might be a result of imbalance between CSF secretion and resorption as has been suggested for ventricular volume changes induced by long-duration spaceflight (Van Ombergen et al., 2019).

In summary, using diffusion imaging we have detected evidence for persistent axonal injury in rats exposed to a single blast in the absence of gross tissue alterations or hemorrhages. Injury severity was not uniform but spatially dependent in both medial-lateral and rostrocaudal directions of the white matter tracts examined. Our results also indicate that diffusion metrics are sensitive to the time lapsed after injury with each metric providing a unique insight into the nature of the axonal pathobiology, as confirmed by EM. The complex interrelationship between diffusion metrics presents a strong case for the measurement of axial and radial diffusivities without which the direction of changes in FA and MD alone would not be conclusive in interpretation. Our results point to the sensitivity of diffusion imaging which may have potential for detecting the temporal pattern of blast-induced brain injury and serve as a noninvasive biomarker in preclinical studies of blast injury progression and effectiveness of therapeutic interventions.

## Summary

Injury to major white matter structures caused by exposure to blast was assessed in rats using diffusion MR imaging. Axonal damage, edema, and damage to myelin sheath persisted 2 weeks after blast exposure. MR findings were validated by EM.

## Supplemental Material

sj-pdf-1-asn-10.1177_1759091420922929 - Supplemental material for Diffusion Tensor Imaging Detects Acute and Subacute Changes in Corpus Callosum in Blast-Induced Traumatic Brain Injury Click here for additional data file. Supplemental material, sj-pdf-1-asn-10.1177_1759091420922929 for Diffusion Tensor Imaging Detects Acute and Subacute Changes in Corpus Callosum in Blast-Induced Traumatic Brain Injury by Palamadai N. Venkatasubramanian, Prachi Keni, Roland Gastfield, Limin Li, Daniil Aksenov, Sydney A. Sherman, JulianBailes III Brian Sindelar, John D. Finan, John Lee, Julian E. Bailes and Alice M. Wyrwicz in ASN Neuro
