# Supplementary material for: Diffusion Tensor Imaging Detects Acute and Subacute Changes in Corpus Callosum in Blast-Induced Traumatic Brain Injury
Source: ASN Neuro. 2020 May 13;12:1759091420922929. doi: 10.1177/1759091420922929 (PMC7238783; doi:10.1177/1759091420922929)
Supplement: sj-pdf-1-asn-10.1177_1759091420922929 - Supplemental material for Diffusion Tensor Imaging Detects Acute and Subacute Changes in Corpus Callosum in Blast-Induced Traumatic Brain Injury [file sj-pdf-1-asn-10.1177_1759091420922929.pdf]

Supplementary Table 1: Significance of changes in diffusion metrics in the left hemisphere of bTBI rats

(Results are from Fisher LSD post hoc comparisons)

LOCATION	FA		MD		AD		RD	
	D1:Control vs bTBI	bTBI: D1 vs D14	D1: Control vs bTBI	bTBI: D1 vs D14	D1: Control vs bTBI	bTBI: D1 vs D14	D1: Control vs bTBI	bTBI: D1 vs D14
**MCC**								
Splenium	0.679662	0.000803	0.090961	0.000001665	0.094956	0.009695	0.501062	0.025062
Body	0.072135	0.006409	0.075607	0.000000085	0.461315	0.002294	0.005334	0.001292
Genu	0.002818	0.007365	0.000086	0.000001024	0.020621	0.016448	0.000760	0.000003
**LCC**								
Splenium	0.000331	0.004761	0.003508	0.000000000	0.032385	0.000054	0.580439	0.981207
Body	0.000443	0.000087	0.000001	0.000000000	0.000203	0.000031	0.206139	0.623793
Genu	0.180501	0.020521	0.030801	0.000000002	0.014017	0.000258	0.728029	0.298201
**CG**								
Caudal	0.000283	0.258446	0.036688	0.000000013	0.161690	0.011764	0.000334	0.000000032
Middle	0.000242	0.027097	0.007561	0.000000000	0.014831	0.001105	0.486280	0.000000176
Rostral	0.016707	0.100008	0.014680	0.000000001	0.089182	0.000023	0.143375	0.000007

**Significance:**	**p<0.01**	**p<0.05**

Supplementary Table 2: Diffusion changes in the right hemisphere of bTBI rats

(Directions of significant diffusion metric changes are shown. Left hemisphere changes followed a similar pattern.)

LOCATION	FA		MD		AD		RD	
	D1: Control vs bTBI	bTBI: D1 vs D14	D1: Control vs bTBI	bTBI: D1 vs D14	D1: Control vs bTBI	bTBI: D1 vs D14	D1: Control vs bTBI	bTBI: D1 vs D14
**Medial CC**								
Splenium		↑		↓		↓		
Body	↓	↑	↑	↓		↓	↑	↑
Genu	↓	↑	↑	↓	↑	↓	↑	↑
**Lateral CC**								
Splenium	↓	↑	↑	↓		↓		
Body	↓	↑	↑	↓		↓		
Genu	↓	↑		↓		↓		
**Cingulum**								
Caudal	↓	↑		↓		↑	↑	↓
Middle	↓	↑	↑	↓		↑		↓
Rostral	↓	↑	↑	↓		↑		↓
